# Prime-pull vaccination with a plant-derived virus-like particle influenza vaccine elicits a broad immune response and protects aged mice from death and frailty after challenge

**DOI:** 10.1186/s12979-019-0167-6

**Published:** 2019-11-04

**Authors:** Breanna Hodgins, Stephane Pillet, Nathalie Landry, Brian J. Ward

**Affiliations:** 1 Department of Experimental Medicine, McGill University, Montreal, Quebec Canada; 2 Research Institute of McGill University Health Centre, 1001 Boul Decarie, Room EM33248, Montreal, QC H4A 3J1 Canada; 3 Medicago Inc., Quebec City, Quebec Canada

**Keywords:** Aged mouse model, Frailty, Influenza vaccines, Virus-like particles, Multi-modality, Prime-pull

## Abstract

**Background:**

Administered intramuscularly (IM), plant-derived, virus-like-particle (VLP) vaccines based on the influenza hemagglutinin (HA) protein elicit both humoral and cellular responses that can protect aged mice from lethal challenge. Unlike split virus vaccines, VLPs can be administered by different routes including intranasally (IN). We evaluated novel vaccine strategies such as prime-pull (IM boosted by IN) and multi-modality vaccination (IM and IN given simultaneously). We wished to determine if these approaches would provide better quality protection in old mice after less severe (borderline-lethal) challenge (ie: immunogenicity, frailty and survival).

**Results:**

Survival rates were similar in all vaccinated groups. Antibody responses were modest in all groups but tended to be higher in VLP groups compared to inactivated influenza vaccine (IIV) recipients. All VLP groups had higher splenocyte T cell responses than the split virus group. Lung homogenate chemokine/cytokine levels and virus loads were lower in the VLP groups compared to IIV recipients 3 days after challenge (*p* < 0.05 for viral load vs all VLP groups combined). The VLP-vaccinated groups also had less weight loss and recovered more rapidly than the IIV recipients. There was limited evidence of an immunologic or survival advantage with IN delivery of the VLP vaccine.

**Conclusion:**

Compared to IIV, the plant-derived VLP vaccine induced a broader immune response in aged mice (cellular and humoral) using either traditional (IM/IM) or novel schedules (multi-modality, prime-pull).

## Background

Influenza infection can be devastating in the elderly, resulting in significant mortality and morbidity [1, 2]. In most seasonal influenza outbreaks, those ≥65 years of age typically account for 71–85% of the deaths that are relatively easy to ‘count’ [3, 4]. The impact of influenza–associated morbidity is more difficult to quantify since even a short period of forced bed-rest, either at home or in hospital, can lead to major loss of muscle mass (ie: sarcopenia) [5] and accumulation of other physiologic and mental deficits (ie: increased frailty) [6, 7]. More prolonged periods of bed-rest (ie: influenza complicated by pneumonia, intensive care admission) often lead to catastrophic disability with loss of independence in elderly subjects [8, 9]. Vaccination is currently the best strategy to protect the elderly from influenza viruses [10] but this population often responds poorly to ‘standard’ inactivated influenza vaccines (IIV) due to prior experience with influenza antigens and immunosenescence [11–13]. Although a number of vaccines that specifically target the elderly have been introduced in recent years (eg: MF59-adjuvanted IIV, high-dose (HD)-IIV) [14], their impact on effectiveness (ie: preventing infection) have been relatively modest [15–20]. Their potential advantages in preventing frailty have generally not been considered.

Several highly-successful virus-like particle (VLP) vaccines are in current use (eg: HBV and HPV vaccines) and VLP vaccines have many potential advantages for a wide range of targets [21–24]. Some VLP vaccines for influenza are at various stages of pre-clinical and clinical development. One of the most advanced is produced by Medicago Inc. (Quebec, QC) using transient production of the influenza hemagglutinin (HA) protein in *Nicotiana benthamiana* plants. After peripheral administration in mice, these plant-derived VLPs move rapidly to regional lymph nodes where they preferentially interact with B cells, NK cells and antigen-presenting cells (APC) [25]. They also interact directly with human immune cells including B cells and APC leading to activation [26], internalization [27] and presentation [28]. Indeed, these plant-derived VLPs appear to recapitulate many of the early interactions of intact influenza virions with host cells including fusion with host endosomal membranes [28]. In animal models of pandemic infection, the plant-derived vaccines can provide excellent protection despite eliciting little-to-no antibody response suggesting an unusual capacity to induce cellular responses [24, 29, 30]. In clinical trials with healthy adults, the plant-derived VLP vaccines not only elicit good antibody levels against seasonal strains but also induce long-lived and poly-functional CD4^+^ T cell responses [29]. The latter characteristic is of particular interest for older individuals since this population may be protected primarily by cellular immunity [31].

In the context of the current work, one major advantage of VLP vaccines is their flexibility: they can be administered using different routes including intramuscular (IM), intradermal (ID), oral (PO) and intranasal (IN) [32, 33]. This flexibility makes alternate vaccination strategies possible including either simultaneous or sequential administration at different sites. The former can be considered a type of multi-modality immunization that, in theory, could stimulate different, tissue-specific immune mechanisms. The latter approach, sometimes referred to as ‘prime-pull’, consists of a systemic “priming” dose (eg: IM) followed by a local “pull” dose given at the site of natural infection to ‘recruit’ antigen-specific immune cells to that area (eg: PO or IN) [34–36]. These alternate vaccination strategies could potentially provide better protection in the elderly by inducing a long-lasting, cross-protective cellular response [37–39] and boosting of local mucosal immunity [34, 40]. As noted above ‘standard’ vaccination strategies based on IM delivery of IIVs that primarily elicit systemic antibodies have had only limited success in the elderly [31, 41]. We were interested to know if the flexibility and unusual immunogenicity of the plant-derived VLP vaccines could be exploited to better protect older individuals. We have recently shown that a single dose of a plant-derived H1-VLP candidate vaccine can protect old mice from a lethal A/California/07/2009 H1N1 challenge [42]. To our surprise, a single dose of the same VLP vaccine administered IN protected ~ 60% of the animals despite the complete absence of a detectable systemic serologic response [42].

In the current work, we extended these observations by testing alternate VLP immunization strategies and following immunogenicity as well as protection against both frailty and death following a borderline-lethal A/California/07/2009 H1N1 challenge. Our results confirmed that the VLP vaccine elicits a broader immune response than IIV regardless of the vaccination strategy used. Animals that received a dose of the VLP vaccine IN had the most rapid weight recovery and the least change in frailty index after challenge infection. Although preliminary, these data suggest that such alternate vaccination strategies should at least be considered for elderly subjects when vaccines with the flexibility to be administered via multiple routes become commercially available.

## Results

### Infection survival rates

**Fig. 1:**
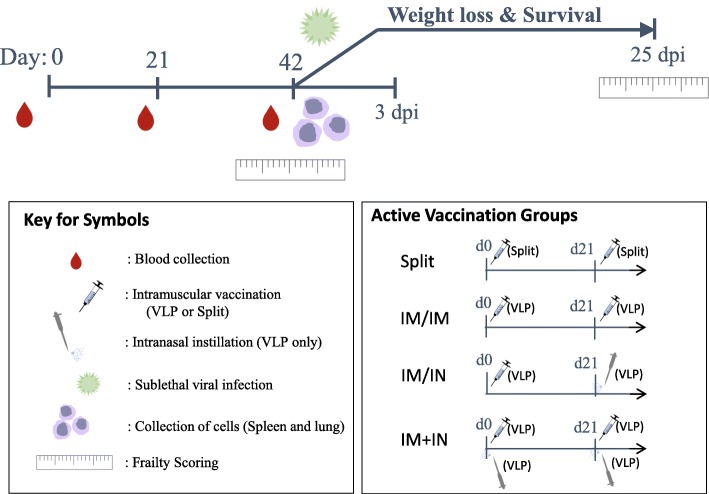
Timeline for vaccine administration. Female BALB/c mice (18–22 months of age) were vaccinated twice on day 0 (d0) and day 21 (d21) with plant-derived H1-VLP vaccine, inactivated H1N1 split vaccine or PBS. Three groups of animals received the VLP vaccine: i) two 3 μg doses intramuscularly (IM/IM) ii) a first 3 μg dose IM boosted at d21 by a 3 μg dose intranasally (IM/IN: Prime-Pull group) or iii) two doses of 1.5 μg IM plus 1.5 μg IN (IM + IN: Multi-modality group). The comparator group received two 3 μg doses of a split inactivated influenza vaccine. Peripheral blood was collected at d0 (pre-vaccination) and at d21 (data not shown) and d42 after the first dose of vaccine. Spleens and lungs were harvested from individual animals. The remaining mice (typically 6–8 mice/group) were scored for frailty on d40–42 then challenged with a sub-lethal dose of wild-type H1N1 A/California/07/2009 virus (525 TCID_50_ in 50 μL) by IN instillation (25 μL/nare). Weight loss was monitored daily for up to 28 days. At d45 or 3 days post-infection (dpi), 3–5 mice/group were sacrificed (isoflurane/CO_2_) and serum (cardiac puncture) and lungs were collected. At d67 (25 ± 4 dpi), surviving mice were scored for frailty and sacrificed to collect serum and lungs

**Fig. 2:**
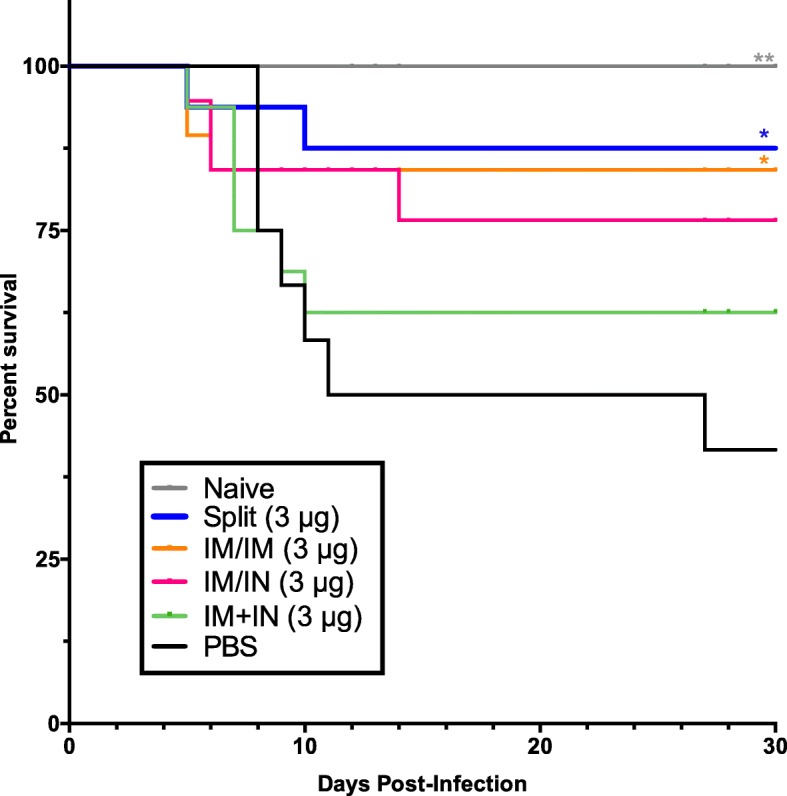
Survival after A/California/07/2009 H1N1 challenge. Aged BALB/c female mice (18–22 months of age) were immunized twice with H1-VLP vaccine or inactivated split vaccine. Six weeks after vaccination, mice were challenged with a sub-lethal dose of A/California/07/2009 H1N1 and were closely monitored for weight loss. Mice were euthanized if they lost > 20% of their initial weight A log-rank (Mantel-Cox) test was used to compare survival curves with the PBS control group (** *p* < 0.01, * *p* < 0.05). This graph is representative of 5–10 mice/group from two separate studies

The viral challenge dose following the vaccination regimen as illustrated in Fig. 1 was intended to be severe but low enough to permit a good proportion of the animals to survive for determination of frailty after infection. The viral inoculum in each replicate experiment was based on titration experiments to permit dosing with ~ 0.5 the TCID_50_ lethal dose. Overall, slightly more than half of the PBS control animals succumbed to infection (41.7% survival) (Fig. 2). The vaccine groups with the highest and significant survival rates were the IIV-IM/IM and VLP-IM/IM recipients (87.5 and 84.2%, respectively). The VLP-IM/IN and VLP-IM + IN groups had a slightly lower survival (76.5 and 62.5%) but these differences did not reach statistically significance. All the naïve, uninfected mice survived.

### Antibody response

**Fig. 3:**
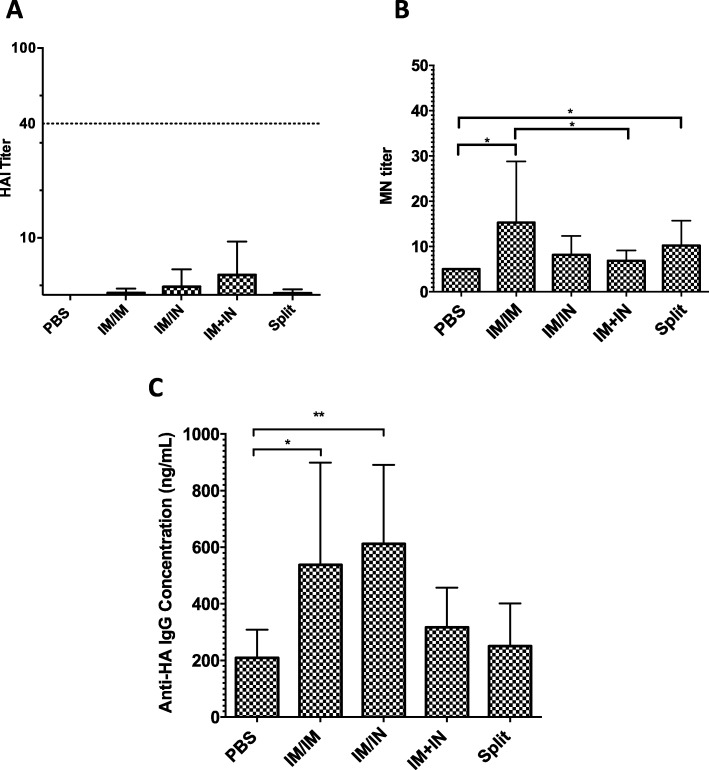
Antibody responses after two vaccinations towards H1N1 A/California/07/2009. Aged BALB/c female mice (18–22 months of age) were immunized twice with H1-VLP or inactivated split vaccine. Six weeks post-vaccination, the humoral response to the H1 of A/California/07/2009 H1N1 was analyzed in sera from individual mice by hemagglutination inhibition (HAI: 8–10 animals/group) (**a**), micronuetralization (MN: 6–8 animals/group) (**b**) and ELISA (**c**: 8–10 animals/group). The dotted line in **a** represents an HAI titre of 1:40, which is considered protective in humans. Error bars indicate 95% CI. For statistical analysis, one-way ANOVA was used on log transformed values (*** *p* < 0.001, ** *p* < 0.01 * *p* < 0.05). These data represent 2 independent studies

Antibody responses in these older mice were generally weak. The highest geometric mean HI titres were observed was the VLP-IM + IN group (GMT: 6.4 ± 4.0) but only 20% of the animals mounted a detectable response (Fig. 3a). Only 5–7% of the animals in the other vaccine groups and none of the PBS control animals had a detectable HI response. Although geometric mean MN responses were slightly higher, only a small number of animals in each vaccine group had detectable responses (25–56%). The highest MN response was in the VLP-IM/IM group (GMT: 15.3 ± 44.54; range 10–160 (Fig. 3b) which was significantly higher than the VLP-IM + IN and placebo groups (GMT:6.8 ± 8.80 and < 5, both *p* < 0.05) The mean GMT in the IIV-IM/IM group was 10.21 (range 5–80) and was significantly greater than only the PBS group (< 5: *p* < 0.05). The ELISA assay demonstrated a more consistent antibody response (53–79% of the animals in vaccinated groups mounted detectable levels). The VLP-IM/IN and VLP-IM/IM groups had the highest ELISA titres (GMT: 611.8 ng/mL; 95% confidence interval (CI): 420–891 ng/mL and GMT: 537.7 ng/mL; 95% CI: 322–899 ng/mL, respectively). These titres were significantly higher than the PBS group (GMT: 209.1 ng/mL; 95% CI: 142–309 ng/mL: both p < 0.05). (Fig. 3c). Lower ELISA responses were seen in the VLP-IM + IN (GMT: 317.04 ng/mL; 95% CI: 220–458 ng/mL) and IIV-IM/IM groups but differences between groups did not reached statistical significance.

### Cellular immune response

#### CD4^+^ T cells

**Fig. 4:**
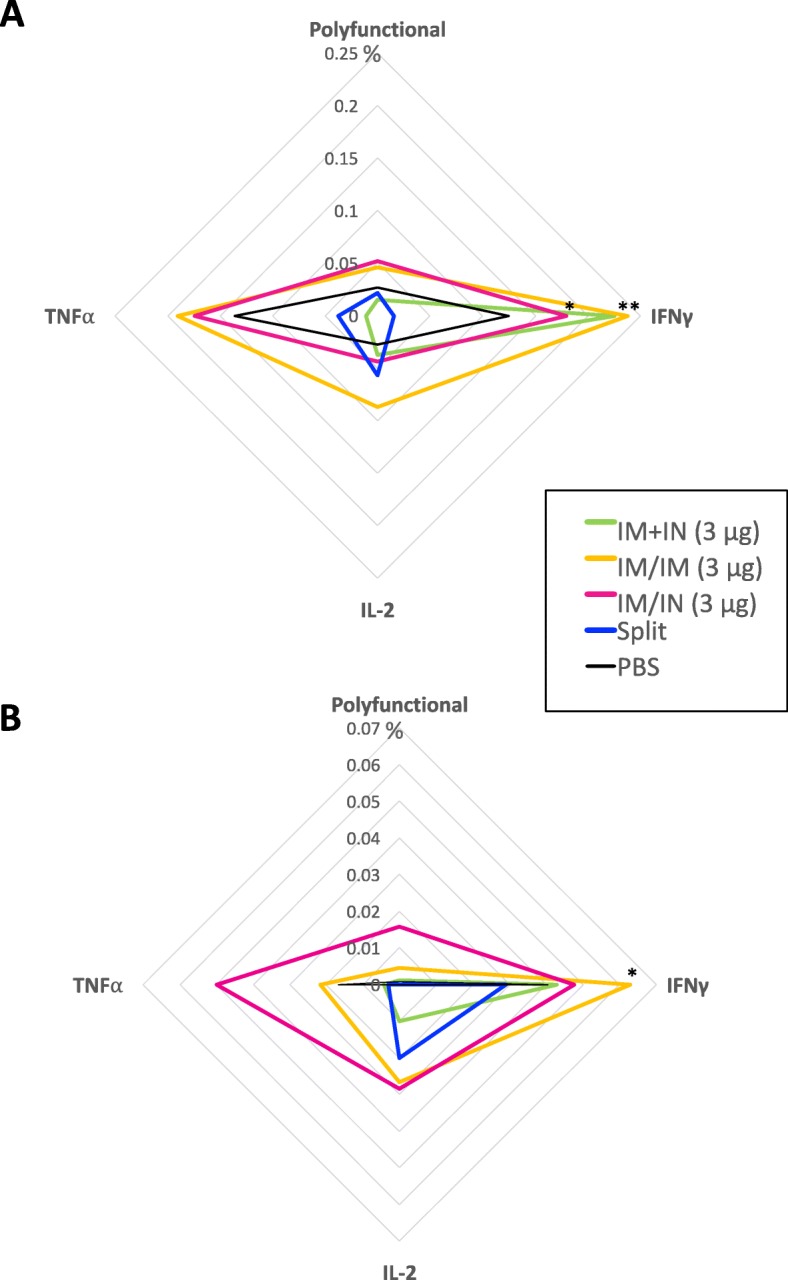
Splenocyte T cells expressing cytokines in response to H1 re-stimulation ex-vivo*.* Splenocytes were collected 3 weeks post-boost from aged female BALB/c mice (18–22 months of age). Percent of splenocytes **a** CD4^+^ T cells and **b** CD8^+^ T cells expressing 2 or more cytokines (polyfunctional) or single cytokines (IFNγ, IL-2 or TNF⍺). For statistical analysis, two-way ANOVA was performed followed by Tukey’s multiple comparison test (**** *p* < 0.0001, *** *p* < 0.001, * *p* < 0.05) compared to the split vaccine. **a** and **b** are representative data from 6 to 8 mice/group from two studies

In contrast to the relatively weak and inconsistent antibody responses seen in the aged mice, HA-specific T cell responses could be detected in most of the animals that had received VLP vaccination, regardless of route or schedule. Overall, 59% of the VLP-vaccinated animals had poly-functional T cell responses above the mean of the PBS animals compared to the 44% of IIV-vaccinated mice (Additional file 1: Table S3). At day 42 post-vaccination, 0.05% of splenocyte CD4^+^ T cells in the VLP-IM/IM and VLP-IM/IN groups had a poly-functional response to H1-VLP stimulation ex vivo that was roughly twice that seen in the VLP-IM + IN (0.02%), IIV-IM/IM (0.02%) and PBS groups (0.03%) (Fig. 4a). Considering single-positive CD4^+^ T cells, H1 antigen-specific responses were most convincingly seen in the VLP-IM/IM and VLP-IM/IN groups (IL-2, TNFα and IFNγ Fig. 4a) but the VLP-IM + IN group also mounted a strong IFNγ response compared to the IIV-IM/IM and PBS groups (*p* < 0.05). Overall, the IIV-IM/IM group did not elicit any CD4^+^ T cell response above the baseline levels seen in the PBS group.

#### CD8^+^ T cells

Antigen-specific CD8^+^ T cell cytokine responses were more variable than CD4^+^ responses but were still more consistently observed in the VLP-vaccinated animals than antibody responses. Overall, poly-functional CD8^+^ T cell responses above mean PBS levels were found in 40% of the VLP-vaccinated animals and 0% of the IIV-vaccinated mice (Additional file 1: Table S3). Again, the VLP-IM/IN and VLP-IM/IM groups had the most convincing CD8^+^ T cell responses; generally, for the VLP-IM/IN groups (poly-functional and all individual cytokines) and for IL-2 and IFNγ in the VLP-IM/IM group (reaching significance for IFNγ versus PBS: *p* < 0.05) (Fig. 4b). Again, the IIV-IM/IM group mounted little-to-no CD8^+^ T cell response above baseline levels except for IL-2 production (0.03% versus 0.001% in the PBS group) although this difference did not reach statistical significance.

### Lung viral loads, histology and cytokine/chemokine concentrations 3 dpi

**Fig. 5:**
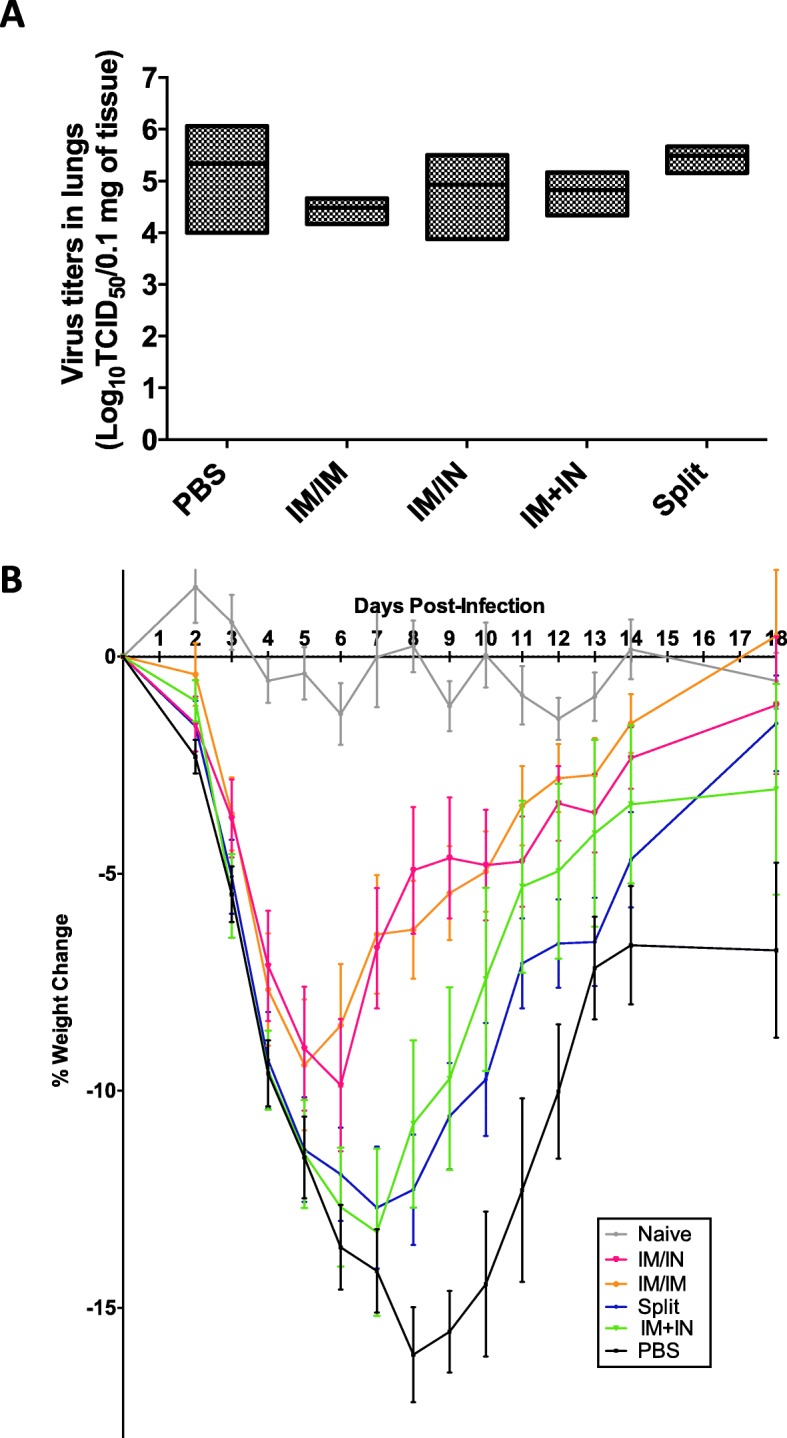
Lung viral loads and weight loss after challenge. Aged BALB/c mice (18–22 months) lungs were collected at 3 days post-infection after sub-lethal challenge with H1N1 A/California/07/2009. Three days post-infection **a** lung viral loads were measured and throughout the infection mice were closely monitored for **b** weight loss. **a** is representative of 3–5 mice/group from two studies and **b** are representative of 5–10 mice/group combined from two studies. For statistical analysis, **a** One-way ANOVA was performed on the log10 values of the viral titres and the Tukey’s multiple comparison test was performed. For **b** Two-way ANOVA followed by the Tukey’s multiple comparison test (**** *p* < 0.0001, *** *p* 0.001, ** *p* < 0.01, * *p* < 0.05) (see Additional file 1: Table S2). Error bars represent the standard error of the mean

**Fig. 6:**
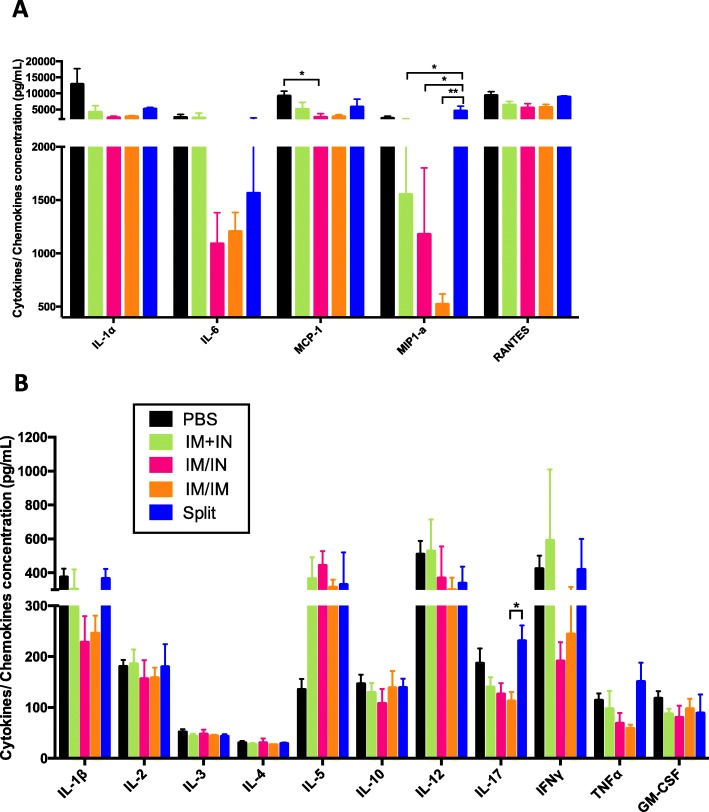
Cytokine and chemokine levels in lung homogenates 3 days after challenge. Six weeks post-vaccination, female BALB/c mice (18–22 months) were challenged with A/California/07/2009 H1N1 and lungs were collected and homogenized 3 days post-infection to measure cytokines/chemokines by multiplex ELISA for the following cytokines: **a** IL-1α, IL-6, MCP-1, MIP-1α, RANTES and **b** IL-1β, IL-2, IL-3, IL-4, IL-5, IL-10, IL-12, IL-17, IFNγ, TNFα, GM-CSF. For statistical analysis, one-way ANOVA followed by Tukey’s multiple comparison test (**p < 0.01, * *p* < 0.05). Data represent 4–7 mice/group from one study. Error bars represent the standard error of the mean

The VLP-IM/IM and VLP-IM/IN vaccinated groups had similar lung viral titres at 3dpi (log_10_ TCID_50_: 4.4 ± 0.3 and 4.5 ± 0.6 respectively) (Fig. 5a). The viral load in the VLP-IM + IN group was slightly higher (4.7 ± 0.3). The highest viral loads were seen in the PBS (4.9 ± 0.6) and the IIV-IM/IM groups (5.4 ± 0.3). When the three groups that received a VLP vaccine were combined (VLP-IM/IM, VLP-IM/IN and VLP-IM + IN) the lung viral load (4.6 ± 0.4) was lower than both the IIV-IM/IM and PBS groups (5.4 ± 0.3 and 4.9 ± 0.6 respectively) but statistical significance was only reached for IIV-IM/IM (*p* < 0.05). Lung histopathology tracked the viral loads quite closely (Table 1). The three VLP groups had the lowest scores (IM/IM: 5.25 ± 3.27, IM/IN:6.00 ± 3.29 and IM + IN: 6.38 ± 3.58). The score of the PBS group was higher (7.83 ± 2.25) and those that had the highest scores were the mice that received the split vaccine (13.25 ± 0.75). Because histopathology scores were only obtained for small numbers of animals in each group, none of these differences reached statistical significance. Cytokine and chemokine concentrations in lung homogenates at 3 dpi tended to be lower overall in the VLP-vaccinated animals (independent of route) than the IIV-IM/IM and PBS groups (Fig. 6). These differences reached statistical significance for MIP1-⍺ between the IIV-IM/IM and all of the VLP groups (p range from < 0.01 to < 0.05) (Fig. 6a) and for IL-17 between the VLP-IM/IM and IIV-IM/IM groups (112.73 pg/mL vs 230.93 pg/mL, respectively, *p* < 0.05) (Fig. 6b). With the exception of low IL-5 levels seen only in the PBS animals, the highest cytokine/chemokine levels tended to occur in the PBS and IIV-IM/IM groups, perhaps as a reflection of the higher lung viral loads and consistent with the greater degree of lung damage.

**Table 1:** Summary of total histopathology scores (20 points total) from H&E stain at 3 days post-infection

	Total Score
PBS	7.83 ± 2.25
Split Vaccine	13.25 ± 0.75
IM/ IM	5.25 ± 3.27
IM + IN	6.00 ± 3.29
IM/IN	6.38 ± 3.58

### Weight loss

Two of the groups that that received VLP vaccines (VLP-IM/IM and VLP-IM/IN) lost the least amount of weight (− 9.4 ± 1.5% and − 9.9 ± 1.5% respectively) and recovered most rapidly, returning to near baseline weights by 18 dpi (Fig. 5b). The PBS control lost the most weight (− 16.1%) and remained well below their baseline weight at 18 dpi (− 6.8 ± 2.0%). The VLP-IM + IN and IIV-IM/IM groups were intermediate in both their maximum weight loss (− 13.3 ± 1.9% and − 12.7 ± 1.4% respectively) and the timing of weight recovery (still − 3.1 ± 2.4% and − 1.5 ± 1.1% at 18 dpi respectively).

### Clinical frailty

**Fig. 7:**
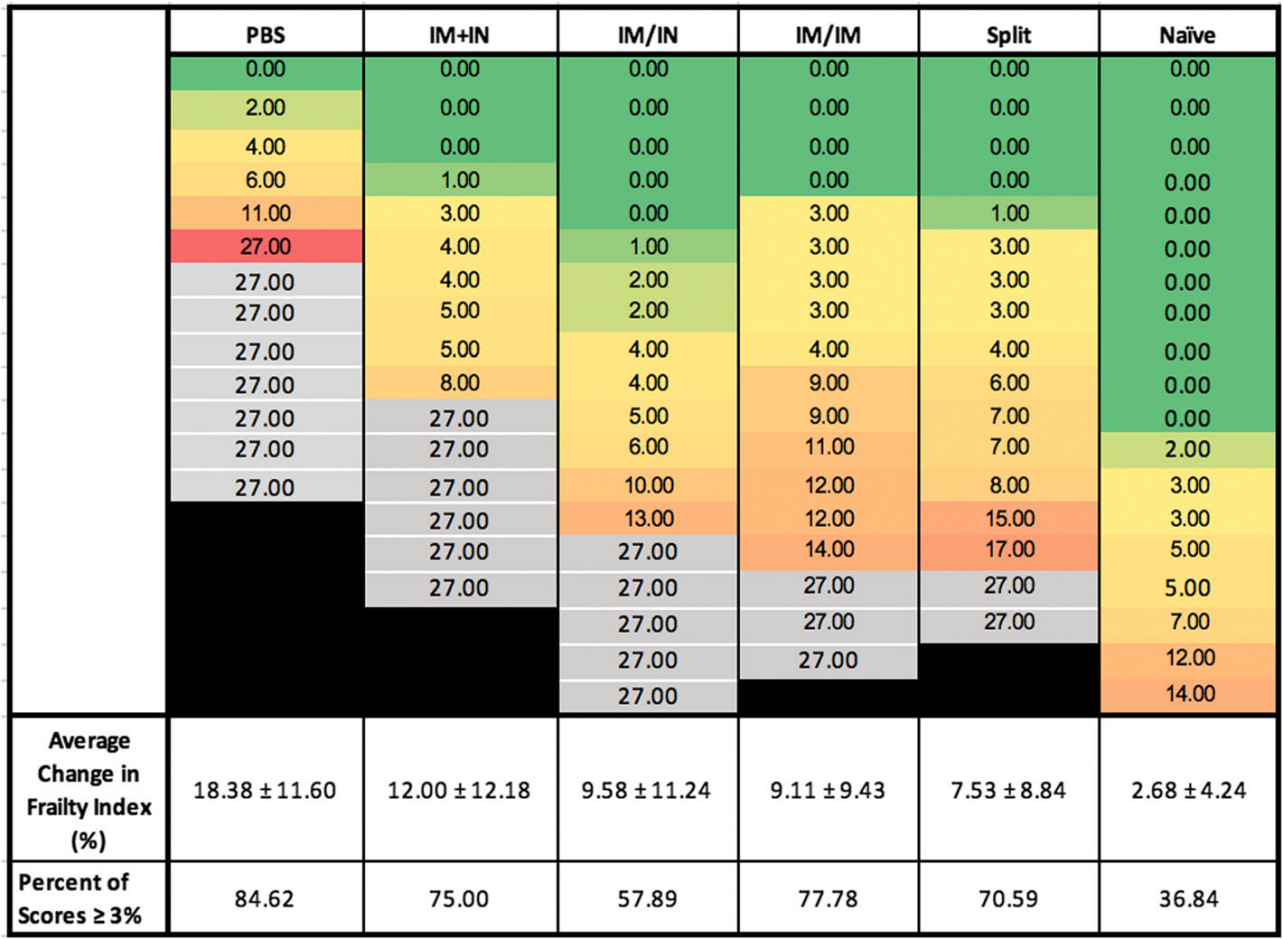
Changes in clinical frailty index after challenge. At day 0 (post-vaccination but pre-challenge) and day 25 post-infection, clinical frailty indices were measured. The percent difference was calculated for each mouse that survived (25 dpi-0 dpi*100). A proportion of those with ≥3% change in each group was also calculated. Assigned frailty changes due to death are outlined in black. For statistical analysis, one-way ANOVA followed by Tukey’s multiple comparison test was used to compared to groups. Data is representative of 6–8 mice/group from 2 studies

Changes in frailty between the time of infection and 25 dpi (± 4 days) are shown in Fig. 7. The overall frailty of these old animals is clear from the fact that the frailty score increased in even the uninfected control group (2.68%). In experiments that included assessment of frailty, the PBS group had the highest mortality (31.6%) and the greatest increase in the frailty index (18.38 ± 11.60%) which was significantly higher than the change in frailty in all of the other vaccinated groups combined (9.51 ± 10.38%; *p* = 0.0156). Overall, 76.9% of the animals that received any vaccine survived. The lowest increases in frailty scores after infection were seen in groups that had received VLP vaccination either IM/IN (9.58 ± 11.24%), IM/IM (9.11 ± 9.43%) or the split IM/IM (7.53 ± 8.84%). Higher frailty scores were seen in the animals that received VLP IM + IN (12.00 ± 12.18%) and this groups had the highest number of deaths (6 animals) compared to all the vaccinated groups (Fig. 7). Although the average frailty scores were the lowest in the split vaccine group, if we assess the scores of the individual mice, we see that those who received the VLP by the multi-modality route had the least number of mice with scores higher than 3% compared to the traditional IM dose (57.89% vs VLP: 77.78% and split: 70.59%). None of the differences between the vaccinated groups reached statistical significance. These findings mirror observations in elderly people that vaccines can protect against both mortality and increases in frailty after infection and, to some extent at least, validate the aged mouse model for this purpose.

## Discussion

The development of better influenza vaccines for the elderly is not only a major problem; it is also a rapidly growing problem as most of industrialized countries of the world continue their epidemiologic transition towards the ‘older end’ of the age spectrum [43]. Immune responses to influenza vaccination in older subjects are subject to a wide range of influences including a life-time of exposures to wild-type viruses as well as vaccines [13, 44] and loss of immune competence due to thymic involution, CMV infection, chronic inflammation and other factors (ie: immunosenescence and/or inflamm-aging) [45–48]. It should therefore be no surprise when vaccines that work reasonably well in children and healthy young adults fail to work in the vulnerable elderly population. This is particularly true since a great deal of effort has been expended to optimize the ability of standard IIVs to elicit antibodies, and specifically antibodies detected by the classical hemagglutination inhibition (HI) assay [41, 49], when it seems increasingly clear that the elderly are protected primarily by other immune mechanisms including T cell responses [31].

New vaccines and vaccination strategies that elicit a different pattern of immunity from that induced by IIVs are clearly needed to provide better protection in older individuals. Among the many novel influenza vaccine candidates moving forward through pre-clinical and clinical testing [50], the plant-derived VLP vaccines that were the focus of the current work have many attractive features. They have considerable flexibility regarding route of administration [42, 51], they are efficiently delivered to lymph nodes and APCs [25–28] and they have been shown to elicit both humoral and cellular responses in animal models and human trials [23, 24, 30, 42, 51]. In addition to new vaccines and strategies, it is also important to use appropriate animal models in developing better influenza vaccines for the elderly [52]. Although ferrets are widely viewed as the best animal model for influenza infection [53], they live for much longer and are much more expensive than mice, not to mention the limited availability of immunologic reagents [54] and their very large teeth. Indeed, we are aware of only a single study of influenza vaccination or infection conducted in aged ferrets [55]. In contrast, aged mice have been used in influenza and influenza vaccine research for at least 40 years due to their relatively short life-spans, their immunologic tractability and their relatively low cost. However, their limitations as models for human elderly should be acknowledged. For example, even very old mice are typically influenza naïve when used in studies rather than having had a life-time of varied exposures to different influenza strains and vaccines [56]. Of course, as with any complex human disease, immune responses in mice are not always fully predictive of response in humans (‘mice lie, monkeys exaggerate’) [57]. Nonetheless, the loss of antibody response despite strong antigen-specific cellular reactivity that we observed in our aged animals following VLP vaccination (Figs. 3 and 4) is certainly consistent with Medicago’s on-going studies of the Quadrivalent influenza VLP vaccine in older subjects (unpublished data). Knowing what to study in aged mice is also critically important. As recently pointed out by Miller et al., we must first ‘know ourselves’ (ie: have a better understanding of the immune correlates of protection) in order to know what kind of immune response we want following vaccination [58]. Continuing this line of reasoning for a moment, it is also important that we know what outcomes to assess. Historically, almost all influenza vaccination studies in aged mice have focused on immunologic parameters (usually just antibody responses as discussed above) and survival. It is very likely that this relatively narrow focus has hampered the development of novel vaccines and vaccination strategies. The recent description of protocols to assess frailty in aged rodent models [59] has been a major advance for influenza vaccine studies. Although complex and time-consuming, the inclusion of frailty assessments in our current work permitted us to recognize subtle differences between vaccines and vaccination schedules that may be highly relevant to protecting the elderly population.

Although some aspects of our work are best considered preliminary, our overall results supported the greater immunogenicity of the VLP vaccine compared to IIV and raised the possibility that delivery of the VLP vaccine IN might have advantages in terms of pattern of immunity generated, lung inflammation and frailty. Although weight loss and mortality among the animals that received any VLP regimen were highest in the VLP-IM + IN group, several other outcomes were very similar between the VLP IM/IN and VLP IM + IN groups including viral loads and histopathology. Lung homogenate cytokine/chemokine profiles were strikingly different in the VLP-IM + IN and VLP-IM/IN animals however (Fig. 6). Despite considerable mouse-to-mouse variability, the VLP-IM + IN animals had much higher levels of pro-inflammatory chemokines/cytokines (IL-Iβ, IL-6, MIP1-α, IFNγ) than the VLP-IM/IN group 3 dpi: a pattern much closer to that seen in the IIV-IM/IM group. Since very little is known about IN dosing of VLP vaccines, a small follow-up study was performed to assess a higher dose of VLPs using the VLP-IM + IN schedule (3 μg/route at each time-point instead of 1.5 μg). Summary data comparing these two doses (Additional file 3: Figure S2) strongly suggest that the 1.5 μg dose used in this study was sub-optimal for IN delivery.

Overall, the conventional IIV-IM/IM strategy was inferior to one or both 3 μg VLP strategies (VLP-IM/IM and VLP-IM/IN) for all outcomes except survival. It is interesting that increasing the dose in the VLP-IM + IN strategy to 3 μg/route not only changed the cytokine/chemokine profile and significantly reduced viral titres at 3 dpi (*p* < 0.01), it also increased survival (84.2%) and dramatically increased lung-resident T cell responses (Additional file 3: Figure S2). Although lung-resident T cells were barely detectable in other groups, the VLP-IM + IN (6 μg) animals had a significantly higher number of lung tissue-resident memory (TRM), antigen-specific CD4^+^ T cells (*p* < 0.01) and a higher proportion (%) of antigen-specific CD8^+^ T cells expressing IFNγ (Additional file 3: Figure S2). TRM CD8^+^ T cells may be critical in viral clearance [38, 60, 61] and TRM CD4^+^ cells are also thought to be important for influenza protection [62, 63]. Indeed, TRM CD4^+^ cells appear to be a good marker for protection from morbidity and mortality in murine models of influenza infection [39, 64]. Moreover, CD4^+^ T cell epitopes are relatively conserved across different influenza strains [65, 66] and TRM CD4^+^ cells may play a particularly important role in protecting against reinfection [39, 64]. Pre-existing, antigen-specific, peripheral blood CD4^+^ T cells have also been shown to be a good correlate of immunity in human challenge studies [19]. It is interesting that an early trial with a live-attenuated H3N2 vaccine by Treanor and colleagues demonstrated better protection in elderly subjects who received simultaneous IIV + attenuated virus IN compared to IIV alone [67]. Although a small number of investigators have pursued prime-pull strategies for seasonal vaccination in children [68] or pandemic vaccines in adults [69, 70], the number of vaccine options has been limited and there have been no further studies in the elderly to our knowledge. Together, these observations and our data strongly suggest that multi-modality or prime-pull strategies may have important advantages for elderly subjects.

## Conclusion

In conclusion, we exploited the flexibility of the VLP vaccine and our aged mouse model to compare the standard vaccine (IIV-IM/IM) and the novel vaccine delivered IM (VLP-IM/IM) as well as multi-modality (VLP-IM + IN) and prime-pull (VLP-IM/IN) strategies. Each of these approaches was assessed using both conventional methods (eg: viral loads, survival curves, classic serologies) as well as less common methods including splenocyte and tissue-resident memory T cell responses, lung cytokine/chemokine profiles and frailty to assess the potential for novel approaches to improve vaccine-induced protection in the elderly. Our findings strongly support the further exploration of such alternative vaccination strategies in older subjects.

## Methods

### Virus, mice and vaccines

Female BALB/c mice (18–22 months of age: Charles River Laboratories, Montreal, QC) were vaccinated twice on day 0 (d0) and day 21 (d21). All active vaccinations were based on hemagglutinin (HA) content for H1N1 A/California/07/2009 (pdmH1N1). The plant-derived H1-VLP vaccine was produced by Medicago Inc. (Quebec City, QC) as previously described [71] using the wild-type HA sequence from pdmH1N1. Three groups of animals received the VLP vaccine: i) two 3 μg doses intramuscularly (IM/IM) ii) a first 3 μg dose IM boosted at d21 by a 3 μg dose intranasally (IM/IN: Prime-Pull group) or iii) two doses of 1.5 μg IM plus 1.5 μg IN (IM + IN: Multi-modality group). The active comparator group received two 3 μg doses of a split inactivated influenza vaccine (H1N1 A/California/07/2009) (IIV: BEI resources, Manassas, VA). Control animals received similar volume IM and/or IN ‘mock’ vaccinations with phosphate buffered saline (PBS: pH:7.4, Wisent, Saint-Bruno, QC). For H1-VLP and PBS injections IM, 50 μL was administered into the right quadriceps muscle using a 28G½ needle. For IIV injections, 50 μL was injected into each quadriceps muscle (100 μL total). Instillations of H1-VLP or PBS IN (25 μL/nare) were performed in lightly isoflurane anesthetized mice (50 μL total).

Peripheral blood was collected using microtainer serum separator tubes (BD Biosciences, Mississauga, ON) from the lateral saphenous vein at d0 (pre-vaccination) and at d21 (data not shown) and d42 after the first dose of vaccine (Fig. 1). Serum was stored at − 20 °C in aliquots until used. At d42 (immediately pre-challenge), approximately ^1^/_2_ of the animals were sacrificed within isoflurane and a CO_2_ chamber (typically 6–8 mice/group). A terminal serum sample was collected by cardiac puncture. Spleens and lungs were harvested from individual animals and processed as described below. The remaining mice (typically 6–8 mice/group) were scored for frailty on d40–42 then challenged with a sub-lethal dose of wild-type H1N1 A/California/07/2009 virus (525 TCID_50_ in 50 μL: National Microbiology Laboratory, Public Health Agency of Canada) by IN instillation (25 μL/nare). Weight loss was monitored daily for up to 28 days. At d45 or 3 days post-infection (dpi), 3–5 mice/group were sacrificed (isoflurane/CO_2_) and serum (cardiac puncture) and lungs were collected (viral load and cytokine analysis). At d67 (25 ± 4 dpi), surviving mice were scored for frailty and sacrificed as above to collect serum and lungs.

### Antibody titre measurements

Serum antibody levels were measured by hemagglutination inhibition assay (HAI), microneutralization assay (MN) and enzyme-linked immunosorbent assay (ELISA) as previously described [42].

### Lung T cell isolation and stimulation

Lungs were perfused with 10 mL of PBS and collected in in Dulbecco’s Modified Eagle’s Medium (DMEM) with 10% FBS (both Wisent, St. Bruno, QC) stored on ice for approximately 2 h before transferring to a lung digestion cocktail (DNAse I (10 mg/mL: Sigma, St. Louis, MO), Collagenase (12.5 mg/mL, Sigma), Liberase (10 mg/mL: Roche, Basel, Switzerland), hyaluronidase I (50 mg/mL, Sigma)) prepared in DMEM. After a 30–40 min incubation at 37 °C in 5% CO_2_, lungs were processed individually through a 70 μm cell strainer, resuspended in 10 mL of Hanks Buffered Salt Solution without calcium/magnesium (HBSS −/−: Wisent) then centrifuged at 320×g for 8 min at 4 °C. Cells were passed through a cell strainer for a second time and washed with 5 mL of HBSS−/− before re-suspension and counting in complete Roswell Park Memorial Institute medium (cRPMI). Lung cells were seeded in duplicate at 1.5 × 10^6^ cells/well in 96-well U-bottom plates (BD Falcon, Mississauga, ON) in 200 μL. Cells were stimulated with cRPMI alone (unstimulated), PMA+ ionomycin (each 1 mg/mL: Sigma) or with 4 μg/mL of a previously-described [24] overlapping H1 peptide pool, from pdmH1N1 (BEI resources, Manassas, VA) (all stimuli at 80 μL/well). Plates were incubated for 5 h at 37 °C in 5% CO_2_.

### Splenocyte isolation and stimulation

Individual spleens were harvested at d42 into HBSS −/− (Wisent) and processed at room temperature (RT) as previously described [72]. Isolated splenocytes were seeded in duplicate in U-bottom 96-well plates (1 × 10^6^ cells/well) as above and stimulated with cRPMI or alone (unstimulated), H1-VLP (2.5 μg/mL HA) or PMA+ ionomycin (each 1 mg/mL: Sigma) for 18 h at 37^°^Cat 5% CO_2_ (all stimuli at 100 μL/well).

### Flow Cytometry

T cell responses was assessed in mononuclear cells isolated from the lungs and spleen at d42 (immediately pre-challenge). Golgi Plug™ (BD Science, San Jose, CA) and added to lung cells at the beginning of stimulation or 13 h after stimulation for splenocytes (20 μL /well). For flow cytometry, cells were washed twice with cold PBS then centrifuged at 320×g, 8 mins at 4 °C. Viability dye (50 μL/well) (Affymetrix ebioscience, Waltham, MA) was added to each well (1:10 for lung cells and 1:100 for splenocytes in PBS) and incubated for 20 min at 4 °C. Cells were washed as above and Fc block (1 μL/well, BD Science, San Jose, CA) was added. The following cocktail was used for surface stains: CD3 –FITC (Clone: 145-2C11, eBioscience), CD4-V500 (Clone: RM4–5, BD Bioscience) and CD8-PerCP-Cy5 (Clone: 53–6.7, BD Bioscience), CD45-BUV495 (Clone: 30-F11, ebioscience), CD11a-APC (Clone: M17/4, Biolegend, San Diego, CA), CD103-BV711 (Clone:M290, BD Bioscience) and CD69-BV605 (Clone:H1.2F3, Biolegend). After 30 min, cells were washed as above, then fixed overnight at 4 °C with 100 μL of fixative (BD Science). For the intracellular stains, cells were washed as above except with 1X permeabilization buffer (BD Science), then stained with an intracellular cocktail containing: IL-2-Pe-Cy5 (Clone: JES6-5H4, Biolegend), IFNγ-PE (Clone: XMG1.2, BD Science) and TNFα-efluor450 (Clone: MP6-XT22, Affymetrix ebioscience) and incubated for 40 min in the dark at 4 °C. After washing with PBS as above, cells with fixed with an intracellular fixative (Affymetrix ebioscience) and analyzed on BD LSRFortessa X-20 (BD Science) using Flowjo software (version 10.0.8r1). Our gating strategy is shown in Additional file 2: Figure S1.

### Frailty measurements

Frailty measurements were adapted from *Whitehead* et al [73] using 29 of the original 31 parameters to adapt the procedure to BALB/c mice. The parameters assessed fell into the following categories: integument, musculoskeletal, ocular and nasal, vestibulocochlear/auditory, digestive, urogenital, respiratory. Signs of discomfort, body weight and temperature were also assessed. Additional file 1: Table S1 is the scoring sheet that was used at d0, d42 and d67 (25 dpi). Deficits were measured using a 3-point scale: 0 = no deficit, 0.5 = mild deficit and 1 = severe deficit. All measurements were performed by the same operators (BH or AB) who were blinded to group assignment. To correct for a survivor effect, animals that died were assigned the highest Frailty Index score of a surviving animal in any group.

### Lung viral load and cytokine/chemokine levels at day 45 (3 dpi)

Both lungs were collected at 3 dpi and homogenized for viral load and cytokine/chemokine measurements as previously described [42, 51, 74]. Briefly*,* viral titres were calculated from the supernatants of lung homogenates using the Karber method and reported as log_10_ 50% tissue culture infectious dose (TCID_50_): logTCID_50_/0.1 mL = − 1 - (observed lysis of monolayer (as a percent(%) /100–0.5) x log10 [75]. Viral load data are representative of 3–5 mice/group from two independent experiments. The lung homogenate supernatants were used (1:5 and 1:10) to measure 16 tissue cytokine/chemokine concentrations using a multiplex ELISA (Quansys, Logan, UT). Lung homogenates were collected from 4 to 7 mice/group in one experiment and tested in duplicate.

### H&E stain

Lung samples from one lung that was excised, fixed and processed for H&E staining as previously described [42]. Briefly, lung samples were fixed in 10% formalin (Fisher Scientific, Ottawa, ON) then embedded in paraffin (Leica, Concord, ON). Sections (4 μm) were applied to slides with a cover slip and scored at 10X and 100X magnification. Slides were scored by a blinded operator (BJW) using a 5 parameter scoring protocol 1) airway epithelial necrosis, attenuation or disruption, 2) airway inflammation, 3) peribronchiolar & perivascular lymphocytic cuffing, 4) alveolar cellular exudate/edema and interlobular edema and 5) alveolar septal inflammatory cells and cellularity [76]. Each parameter was scored from 0 to 4 for a total possible score of 20.

### Statistical analysis

The geometric mean ratios between groups and their 95% confidence intervals (CI) were calculated. For statistical analysis, one-way ANOVA was performed on HAI, ELISA, MNs, viral titres and frailty scores. For survival statistics, a log-rank (Mantel-Cox) test was used. All other statistical analyses used two-way ANOVA. All analyses were performed using GraphPad Prism 6.0 software.

## Supplementary information

**Additional file 1: Table S1.** Frailty Index measurements for day 0 and day 25 post-infection. **Table S2.** Statistics comparing groups for weight loss, using Tukey’s multiple comparison test. **Table S3.** Percent of mouse splenocyte T cells (CD4^+^ or CD8^+^) above the PBS average compared to the vaccinated animals

**Additional file 2:** **Figure S1.** Flow cytometry-gating strategy for splenocytes. Aged (18–22 months) BALB/c mice were immunized twice with H1-VLP, split-virion vaccine or naïve. Three weeks post-boost (6–24 mice/group), splenocytes were collected and stimulated ex vivo for 18 h with H1-VLP.

**Additional file 3:** **Figure S2.** Higher dose IM + IN vaccination: Effects on lung immune response 42 days post-vaccination, viral load 3 dpi and survival after A/California/07/2009 challenge. Aged (18–22 months) BALB/c mice were immunized twice with H1-VLP, split-virion vaccine or PBS. Lungs were collected six weeks after vaccination. Percent of lung A) CD8^+^ T cells and expressing IFNγ presented as background subtracted (stimulated - unstimulated). Fold-change of tissue-resident B) CD4^+^ T cells from the PBS group. For statistical analysis, two-way ANOVA was performed followed by Tukey’s multiple comparison test (** *p* < 0.01, * *p* < 0.05). Six weeks after vaccination, mice were challenged with a sub-lethal dose of A/California/07/09 H1N1 and were closely monitored for weight loss. Three days post-infection C) lung viral loads were measured. D) Survival curve: mice were euthanized if they lost > 20% of their initial weight. A log-rank (Mantel-Cox) test was used to compare survival curves with the PBS control group. (** *p* < 0.01, * *p* < 0.05 compared to naïve group). Data is representative 5–10 mice/group from 2 studies. Error bars represent the standard error of the mean.

## Data Availability

The datasets generated and/or analyzed during the current study are available from the corresponding author on reasonable request.

## Abbreviations

CD: Cluster of differentiation
CO_2_: Carbon dioxide
cRPMI: complete Roswell Park Memorial Institute medium
CTL: Cytotoxic T lymphocytes
d0/3/21/42: days
DMEM: Dulbecco’s modified eagle’s medium
DNAse: Deoxyribonuclease
dpi: days post-infection
ELISA: Enzyme-linked immunosorbent assay
EMEM: Eagle’s minimum essential medium
FBS: Fetal bovine serum
Fc: Fragment, crystallizable
FI: Frailty index
FITC: Fluorescein isothiocyanate
g: gram
GM-CSF: Granulocyte-macrophage colony-stimulating factor
GMT: Geometric mean titer
H & E: Hematoxylin and eosin
HA: Hemagglutinin
HAI: Hemagglutination inhibition assay
HBSS−/−: Hank’s balanced salt solution without calcium or magnesium
HI: Hemagglutination inhibition
IFN: *Interferon*
Ig: Immunoglobulin
*IIV*: *Inactivated influenza vaccine*
*IL*: *Interleukin*
IM: Intramuscular
IN: Intranasal
LD_50_: Lethal dose 50
Log: Logarithm
M: Molar
MCP-1: Monocyte chemoattractant protein 1
mg: milligram
MIP-1α: Macrophage inflammatory protein 1 alpha
mL: milliliter
mLD50: mouse lethal dose 50
mM: millimolar
MN: Microneutralization
NA: Neuraminidase
ng: nanogram
NK: Natural killer cell
nm: nanometer
OD: Optical density
PBS: Phosphate-buffered saline
Pdm H1N1: Pandemic H1N1
PE: Phycoerythrin
PerCP: Peridinin chlorphyll protein
pg: picogram
pH: potential hydrogen
PMA: Phorbol 12-myristate 13-acetate
RANTES: Regulated on activation, normal T cell expressed and secreted
RPMI: Roswell Park Memorial Institute medium
TCID50: 50% Tissue culture infective dose
TNFa: Tumor necrosis factor alpha
TRMs: Tissue resident memory T cells
VLP: Virus-Like-particle
vs: versus
xg: Times gravity
μg: microgram
μL: microliter
μm: micrometer
