# Supplementary material for: Prime-pull vaccination with a plant-derived virus-like particle influenza vaccine elicits a broad immune response and protects aged mice from death and frailty after challenge
Source: Immun Ageing. 2019 Nov 4;16:27. doi: 10.1186/s12979-019-0167-6 (PMC6829930; doi:10.1186/s12979-019-0167-6)
Supplement: Supplementary file 1 — Additional file 1: Table S1. Frailty Index measurements for day 0 and day 25 post-infection. Table S2. Statistics comparing groups for weight loss, using Tukey’s multiple comparison test. Table S3. Percent of mouse splenocyte T cells (CD4+ or CD8+) above the PBS average compared to the vaccinated animals [file 12979_2019_167_MOESM1_ESM.docx]

**Supplemental Figure Legends and Tables**

**Table S1 Frailty Index measurements for day 0 and day 25 post-infection**

**Table S2 Statistics comparing groups for weight loss, using Tukey’s multiple comparison test**

|  |  | PBS | IM+IN | IM/IN | IM/IM | Split | Naïve |
| --- | --- | --- | --- | --- | --- | --- | --- |
| **3 days post-infection** | **PBS** |  |  |  |  |  |  |
|  | **IM+IN** |  |  |  |  |  |  |
|  | **IM/IN** |  |  |  |  |  |  |
|  | **IM/IM** |  |  |  |  |  |  |
|  | **Split** |  |  |  |  |  |  |
|  | **Naïve** | ****** | ****** |  |  | ****** |  |
| **4 days post-infection** | **PBS** |  |  |  |  |  |  |
|  | **IM+IN** |  |  |  |  |  |  |
|  | **IM/IN** |  |  |  |  |  |  |
|  | **IM/IM** |  |  |  |  |  |  |
|  | **Split** |  |  |  |  |  |  |
|  | **Naïve** | ******** | ******** | ****** | ******* | ******** |  |
| **5 days post-infection** | **PBS** |  |  |  |  |  |  |
|  | **IM+IN** |  |  |  |  |  |  |
|  | **IM/IN** |  |  |  |  |  |  |
|  | **IM/IM** |  |  |  |  |  |  |
|  | **Split** |  |  |  |  |  |  |
|  | **Naïve** | ******** | ******** | ******** | ******** | ******** |  |
| **6 days post-infection** | **PBS** |  |  |  |  |  |  |
|  | **IM+IN** |  |  |  |  |  |  |
|  | **IM/IN** |  |  |  |  |  |  |
|  | **IM/IM** | ***** |  |  |  |  |  |
|  | **Split** |  |  |  |  |  |  |
|  | **Naïve** | ******** | ******** | ******** | ******* | ******** |  |
| **7 days post-infection** | **PBS** |  |  |  |  |  |  |
|  | **IM+IN** |  |  |  |  |  |  |
|  | **IM/IN** | ******** | ******* |  |  |  |  |
|  | **IM/IM** | ******** | ******** |  |  |  |  |
|  | **Split** |  |  | ****** | ******* |  |  |
|  | **Naïve** | ******** | ******** | ****** | ****** | ******** |  |
| **8 days post-infection** | **PBS** |  |  |  |  |  |  |
|  | **IM+IN** | ***** |  |  |  |  |  |
|  | **IM/IN** | ******** | ****** |  |  |  |  |
|  | **IM/IM** | ******** |  |  |  |  |  |
|  | **Split** |  |  | ******** | ******* |  |  |
|  | **Naïve** | ******** | ******** | ***** | ****** | ******** |  |
| **9 days post-infection** | **PBS** |  |  |  |  |  |  |
|  | **IM+IN** | ***** |  |  |  |  |  |
|  | **IM/IN** | ******** | ***** |  |  |  |  |
|  | **IM/IM** | ******** |  |  |  |  |  |
|  | **Split** |  |  | ****** | ****** |  |  |
|  | **Naïve** | ******** | ******** | ******** | ******* | ******** |  |
| **10 days post-infection** | **PBS** |  |  |  |  |  |  |
|  | **IM+IN** | ****** |  |  |  |  |  |
|  | **IM/IN** | ******** |  |  |  |  |  |
|  | **IM/IM** | ******** |  |  |  |  |  |
|  | **Split** |  |  | ***** | ***** |  |  |
|  | **Naïve** | ******** | ******* |  | ***** | ******** |  |
| **11 days post-infection** | **PBS** |  |  |  |  |  |  |
|  | **IM+IN** | ***** |  |  |  |  |  |
|  | **IM/IN** | ****** |  |  |  |  |  |
|  | **IM/IM** | ******** |  |  |  |  |  |
|  | **Split** |  |  |  |  |  |  |
|  | **Naïve** | ******** |  |  |  | ****** |  |
| **12 days post-infection** | **PBS** |  |  |  |  |  |  |
|  | **IM+IN** |  |  |  |  |  |  |
|  | **IM/IN** | ***** |  |  |  |  |  |
|  | **IM/IM** | ****** |  |  |  |  |  |
|  | **Split** |  |  |  |  |  |  |
|  | **Naïve** | ****** |  |  |  | ***** |  |
| **13 days post-infection** | **PBS** |  |  |  |  |  |  |
|  | **IM+IN** |  |  |  |  |  |  |
|  | **IM/IN** |  |  |  |  |  |  |
|  | **IM/IM** |  |  |  |  |  |  |
|  | **Split** |  |  |  |  |  |  |
|  | **Naïve** |  |  |  |  | ***** |  |
| **14 days post-infection** | **PBS** |  |  |  |  |  |  |
|  | **IM+IN** |  |  |  |  |  |  |
|  | **IM/IN** |  |  |  |  |  |  |
|  | **IM/IM** |  |  |  |  |  |  |
|  | **Split** |  |  |  |  |  |  |
|  | **Naïve** | ***** |  |  |  |  |  |

**Table S3 Percent of mouse splenocyte T cells (CD4^+^ or CD8^+^) above the PBS average compared to the vaccinated animals.**

|  | *CD4^+^ T cells (%)* | | *CD8^+^ T cells (%)* | | |
| --- | --- | --- | --- | --- | --- |
|  | ***VLP*** | ***IIV*** | ***VLP*** | ***IIV*** |  |
| *Polyfunctional* | 59 | 44 | 40 | 0 |  |
| *IFNγ* | 53 | 0 | 35 | 33 |  |
| *IL-2* | 64 | 89 | 65 | 56 |  |
| *TNFɑ* | 30 | 0 | 38 | 22 |  |
| *Sum of Total (expressing 1 or more cytokines)* | 69 | 11 | 47 | 44 |  |
