# Supplementary figures and images for: Prime-pull vaccination with a plant-derived virus-like particle influenza vaccine elicits a broad immune response and protects aged mice from death and frailty after challenge
Source: Immun Ageing. 2019 Nov 4;16:27. doi: 10.1186/s12979-019-0167-6 (PMC6829930; doi:10.1186/s12979-019-0167-6)

## Slide 1
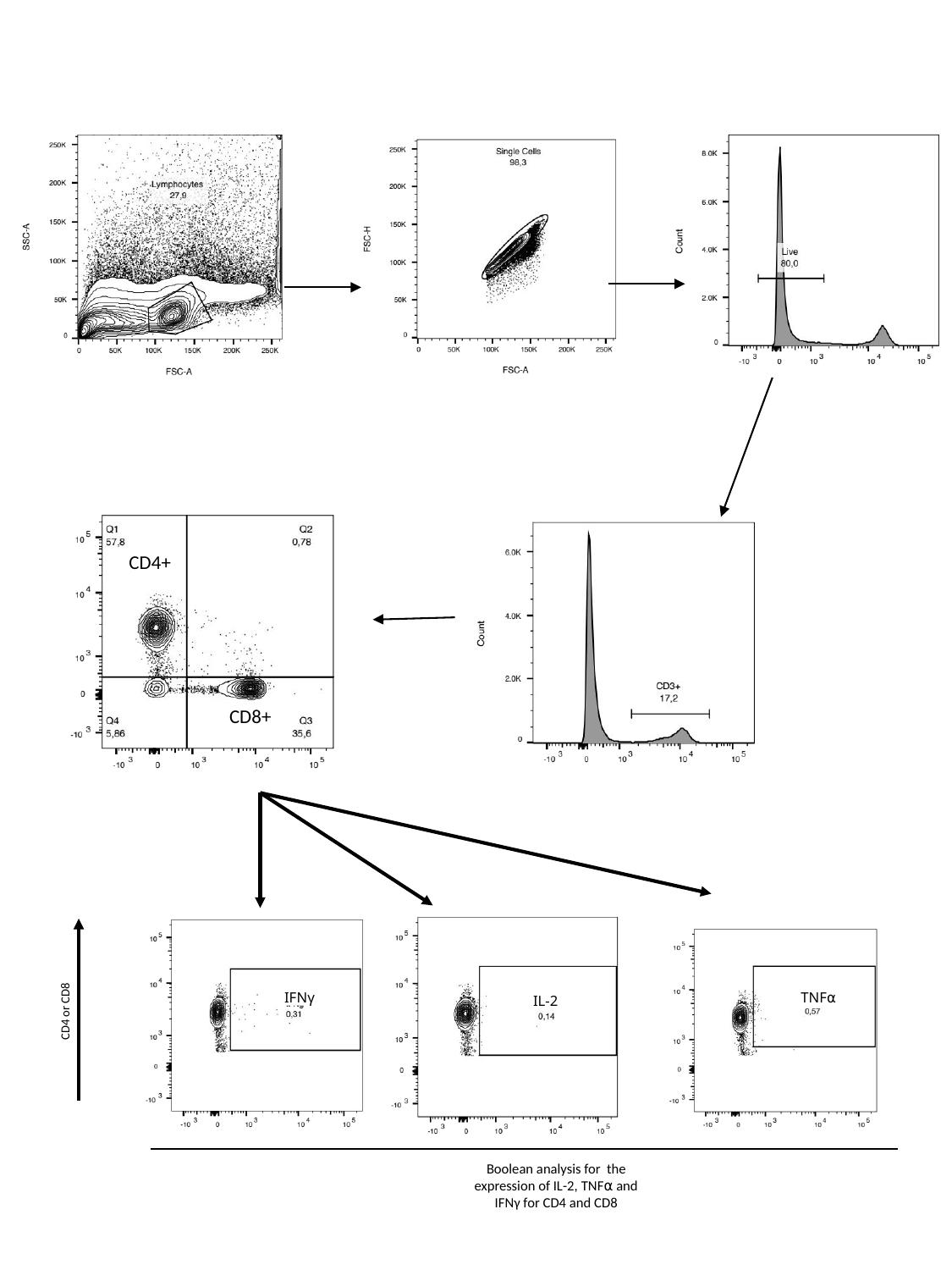

CD4+
CD8+
IFNγ
TNF⍺
IL-2
CD4 or CD8
Boolean analysis for the expression of IL-2, TNF⍺ and IFNγ for CD4 and CD8

Supplement: Supplementary file 2 — Additional file 2: Figure S1. Flow cytometry-gating strategy for splenocytes. Aged (18–22 months) BALB/c mice were immunized twice with H1-VLP, split-virion vaccine or naïve. Three weeks post-boost (6–24 mice/group), splenocytes were collected and stimulated ex vivo for 18 h with H1-VLP. [file 12979_2019_167_MOESM2_ESM.pptx]

## Slide 1
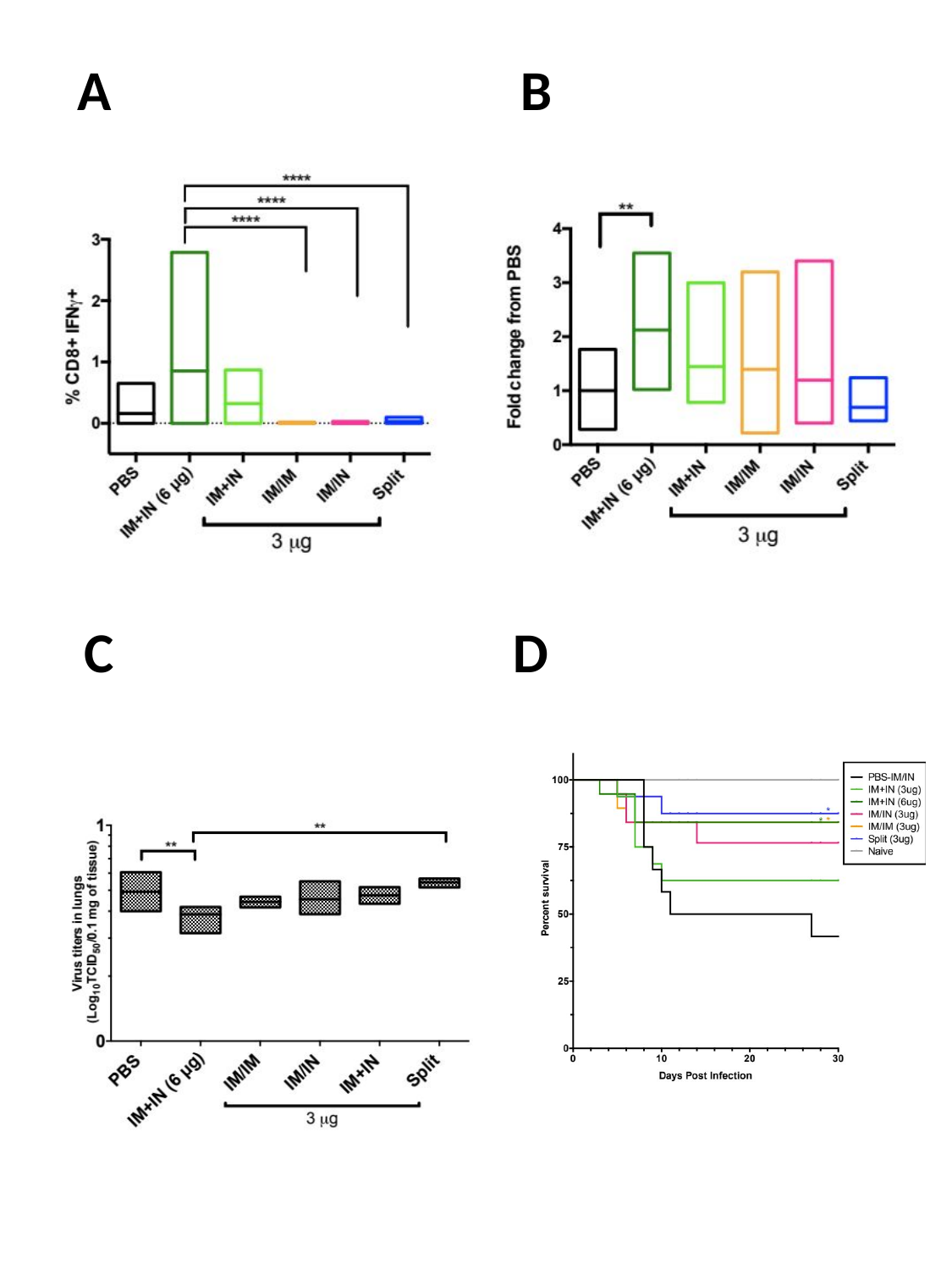

A
B
C
D

Supplement: Supplementary file 3 — Additional file 3: Figure S2. Higher dose IM + IN vaccination: Effects on lung immune response 42 days post-vaccination, viral load 3 dpi and survival after A/California/07/2009 challenge. Aged (18–22 months) BALB/c mice were immunized twice with H1-VLP, split-virion vaccine or PBS. Lungs were collected six weeks after vaccination. Percent of lung A) CD8+ T cells and expressing IFNγ presented as background subtracted (stimulated - unstimulated). Fold-change of tissue-resident B) CD4+ T cells from the PBS group. For statistical analysis, two-way ANOVA was performed followed by Tukey’s multiple comparison test (** p < 0.01, * p < 0.05). Six weeks after vaccination, mice were challenged with a sub-lethal dose of A/California/07/09 H1N1 and were closely monitored for weight loss. Three days post-infection C) lung viral loads were measured. D) Survival curve: mice were euthanized if they lost > 20% of their initial weight. A log-rank (Mantel-Cox) test was used to compare survival curves with the PBS control group. (** p < 0.01, * p < 0.05 compared to naïve group). Data is representative 5–10 mice/group from 2 studies. Error bars represent the standard error of the mean. [file 12979_2019_167_MOESM3_ESM.pptx]
